# The Influence of pH on the Melamine-Dimethylurea-Formaldehyde Co-Condensations: A Quantitative ^13^C-NMR Study

**DOI:** 10.3390/polym9030109

**Published:** 2017-03-17

**Authors:** Ming Cao, Taohong Li, Jiankun Liang, Guanben Du

**Affiliations:** 1 The Yunnan Province Key Lab of Wood Adhesives and Glued Products, Southwest Forestry University, Kunming 650224, China; caominghappy@163.com (M.C.); dushimensheng@126.com (J.L.); 2 College of Materials Science and Engineering, Nanjing Forestry University, Nanjing 210037, China; 3 The Key Laboratory of Forest Resources Conservation and Use in the Southwest Mountains of China, Southwest Forestry University, Ministry of Education, Kunming 650224, China

**Keywords:** co-condensation, melamine-dimethylurea-formaldehyde, ^13^C-NMR

## Abstract

1,3-dimethylurea (DMU) was used to mimic urea and to model melamine-urea-formaldehyde (MUF) co-condensation reactions. The products of 1,3-dimethylurea-formaldehyde (DMUF), melamine-formaldehyde (MF), and melamine-1,3-dimethylurea-formaldehyde (MDMUF) reactions under alkaline and weak acidic conditions were compared by performing quantitative carbon-13 nuclear magnetic resonance (^13^C-NMR) analysis. The effect of pH on the co-condensation reactions was clarified. With the presence of the methyl groups in DMU, the appearance or absence of the featured signal at 54–55 ppm can be used to identify the co-condensed methylene linkage –N(–CH_3_) –CH_2_–NH–. Under alkaline condition, MDMUF reactions produced primarily MF polymers and the featured signal at 54–55 ppm was absent. Even though the co-condensations concurrently occurred, undistinguishable and very minor condensed structures with ether linkage were formed. Differently, under weak acidic condition, the relative content of co-condensed methylene carbons accounts for over 40%, indicating the MDMUF co-condensation reactions were much more competitive than the self-condensations. The formation of reactive carbocation intermediate was proposed to rationalize the results.

## 1. Introduction

Urea-formaldehyde (UF) is one of the major wood adhesive used in wood-based manufactures, such as particleboards, plywoods, medium density fiberboards (MDF), and other non-structural wood products [1]. However, formaldehyde emission from wood composite board and poor resistance to hydrolytic degradation of UF polymers have stimulated efforts to develop improved and/or new adhesives based on UF resins. The common approach to reduce formaldehyde emission is lowering the overall formaldehyde/urea (F/U) molar ratio. However, the bonding strength panel is also sacrificed when lowering the F/U molar ratio. In contrast, melamine-formaldehyde (MF) resin is provided with better water resistant performance and lower level of formaldehyde emission. Therefore, incorporation of melamine in synthesis to improve the properties of UF has become an effective method [1].

However, MUF synthesis is faced with a contradiction. Generally, the UF condensation reactions occurred with an acceptable rate at pH = 4.0–5.5, whereas MF reactions are controllable at pH = 6–10, and generally synthesized at pH above 8. Despite such a contradiction, many efforts have been made to obtain MUF resins. According to whether or not the co-condensation step experiences an acidic stage, the procedures can be divided into two patterns. By employing traditional UF synthesis procedure, the MUF resin can be synthesized using so-called alkaline-acidic procedure [2,3,4,5,6,7,8,9,10,11,12]. In this procedure, a certain amount of melamine can be added together with urea at the initial alkaline stage or added at the following acidic (strong or weak) stage. In another procedure, melamine, urea, and formaldehyde were reacted under alkaline conditions [13,14,15,16,17,18,19]. It was found that, many factors, including the molar ratio of the reactants, pH, amount of added melamine, and adding stage, etc., may affect the performance of the MUF resins. However, it is undisputed that whether or not the co-condensation reactions occurred is the primary issue. To clarify this issue, the MUF resin structures were characterized by using analytical techniques, such as mass spectroscopy (MS) and nuclear magnetic resonance (NMR) [2,3,4,13,16]. The molecular weight distribution of the polymers can be obtained by MS analysis, but the existence of isomers make it difficult to distinguish MUF co-polymers from UF and MF self-condensed polymers. Further, it is almost impossible to make quantitative analyses using MS on the resin, which is a mixture of many different components. In contrast, NMR, especially ^13^C-NMR, was shown to be a powerful tool in quantitatively describing the structure of a resin because the relative contents of different chemical structures can be obtained. However, on the issue of MUF, ^13^C-NMR also encounters challenges. Although it was claimed in some literatures that the co-condensed structures exhibited different ^13^C-NMR signals from self-condensed structures, the evidence is not convincing because the difference of ^13^C-NMR signals between self- and co-condensed structures is generally smaller than 1 ppm, while the NMR peaks are generally broad. Specifically, MUF NMR signals overlap significantly with those of UF and MF. Therefore, it was concluded by Kim et al. that it is difficult to say the co-condensation reactions occurred or not [4]. To find out the evidence of co-condensation reactions, Philbrook et al. analyzed the MUF polymers with correlated ^1^H-^15^N-NMR spectra [20]. From the obtained spectra, the more convincing evidence was found, indeed, but the MUF reactions were investigated at only pH = 7.0. The quantitative results at different pHs were not obtained and compared, so that the effects of pH on the competitive relationship between self- and co-condensations remain unclear.

To overcome the difficulties in distinguish co-condensed structures from self-condensed structures, using model compounds is a good choice. It has been proved that 1,3-dimethylurea (DMU) is an ideal compound and its reactions with formaldehyde can be used to mimic the urea-formaldehyde reactions [21]. With the presence of two methyl groups, the co-condensed structures may exhibit different spectroscopic signals and allow us to identify them. Motivated by this, in this study DMU was used to model the MUF reactions. The aim of this study is not to establish a method of identifying the co-condensed structure, but to clarify the required pH condition for occurrence of MUF co-condensation reactions.

## 2. Experimental

### 2.1. Preparation of the Samples

#### 2.1.1. Samples for DMU-Formaldehyde (DMUF) Reactions under Alkaline and Weak Acidic Conditions

To obtain the desired formaldehyde to DMU molar ratio (F/DMU) of 1/1, 17.96 g of DMU (molecular formula C_3_H_8_N_2_O, purity >98%, J and K Scientific Ltd., Beijing, China) and 16.22 g of the formaldehyde aqueous solution (37%, wt %) were used. Before mixing them, the pH of the formaldehyde solution was adjusted to 9.0–9.5 with 10% NaOH. After the DMU was dissolved completely, the mixture was charged into a flask with a stirring device and a condenser. The solution was then heated to 70 °C in a water bath and the temperature was maintained for 1 h. During this process, the pH was re-adjusted once the drop was observed and maintained in the range of 9.0–9.5. After the reaction was completed, a sample was taken and marked as sample A1.

With similar procedures, the reactions at pH = 6.0–6.3 was carried out. This sample was marked as samples A2.

#### 2.1.2. Samples for Melamine-Formaldehyde (MF) Reactions under Weak Acidic Conditions

The pH of 16.22 g formaldehyde aqueous solution (37%) was pre-adjusted to 6.0–6.3. To avoid fast gelling, 30.66 g of distilled water was added, and then 12.6 g melamine (AR) was charged to obtain the F/M molar ratio of 2/1. The mixture was charged into a flask with a stirring device with a condenser and the pH was re-adjusted to 6.0–6.3. The solution was then heated to 70 °C in a water bath and the temperature was maintained for 1 h. During this process, the pH was maintained in the range of 6.0–6.3. After the reaction was completed, a sample was taken and marked as sample B.

#### 2.1.3. Samples for Melamine-DMU-Formaldehyde (MDMUF) Reactions under Alkaline and Weak Acidic Conditions

With similar procedures, 12.6 g melamine, 17.96 g DMU (purity > 98%), and 16.22 g formaldehyde solution (37%) were mixed and the F/DMU/M molar ratio was 1/1/0.5. The mixture was heated to 70 °C in water bath and the temperature was maintained for 1 h at pH = 9–9.5 and 6.0–6.3, respectively, and the samples that were taken after 1 h were marked as sample C1 and C2, respectively.

### 2.2. ^13^C-NMR Characterizations

The ^13^C nuclear magnetic resonance (^13^C-NMR) spectra were measured using a Brucker AVANCE 600 spectrometer (Bruker Corporation, Billerica, MA, USA). The ^13^C-NMR samples were prepared by mixing 200 μL liquid samples with 200 μL DMSO-d6. The spectra were recorded with a pulse angle of 90 degrees (12 μs) and a relaxation delay of 6 s. To obtain the quantitative results, the inverse-gated decoupling method was applied by using the “zgig” pulse program. The spectra were taken at 150 MHz with 400–600 scans accumulated.

Peak areas from methylene carbons were integrated and summed. Finally, the relative contents of all methylene carbons were calculated as the ratio of the integral value of each type of methylene carbon over the total value of all methylene carbons. The regions of carbonyl carbons in DMU and triazinane carbons in melamine may reflect the substitutions on amino groups, but the peaks appeared to be broader and serious overlap occurred and, therefore, the relative contents of these carbons were not calculated. In addition to this, the carbon at 50 ppm that belongs to methanol and the methyl groups on DMU were not considered in the quantitative analysis because they are not directly involved in the reactions.

To distinguish the methylene carbon at 54–55 ppm from the methyl carbon in –O–CH_3_ structure, the distortionless enhancement by polarization transfer (DEPT) analysis was also performed when necessary. The DETP experiment is typically used for CH_n_ multiplicity determination. In a spectrum of ^13^C-DEPT135, the CH and CH_3_ carbons appear as positive peaks, whereas the CH_2_ carbons appear as negative (inverted) peaks. The quaternary carbons do not appear.

## 3. Results and Discussion

### 3.1. The DMU-Formaldehyde (DMUF) Reactions

Urea has theoretical functionality of four, but the tetra-substituted species have never been isolated, or they are formed but the concentration is too low to be detected. Instead, functionality of three, at most, has been widely accepted; namely, urea can react with up to three units of formaldehyde per molecule. In the typical procedure of UF resin synthesis, the molar ratio of formaldehyde to urea (F/U) the initial alkaline stage is generally controlled to be around 2/1. Consequently, DMU should have a functionality of one and, therefore, a 1/1 molar ratio was used in this study. In the UF resin synthesis, the temperature is generally controlled to be 80–90 °C, but significant hydrolysis of DMU may occur at such temperatures. Therefore, the reaction temperature was set to be 70 °C here.

**Figure 1:**
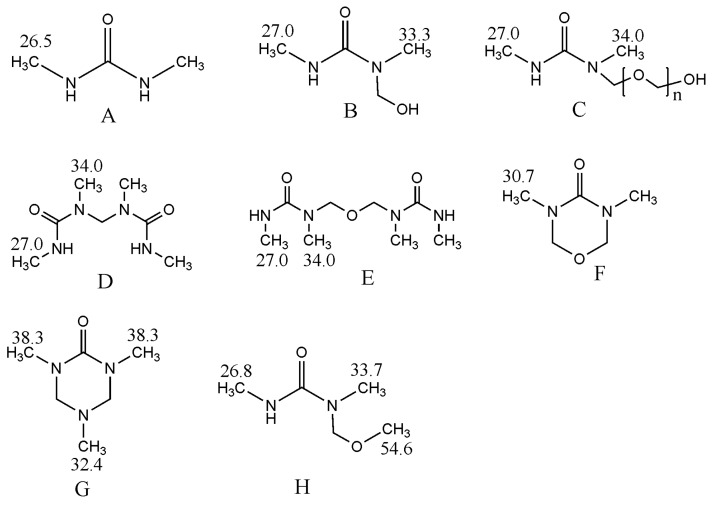
The possible products formed in DMUF reactions.

**Figure 2:**
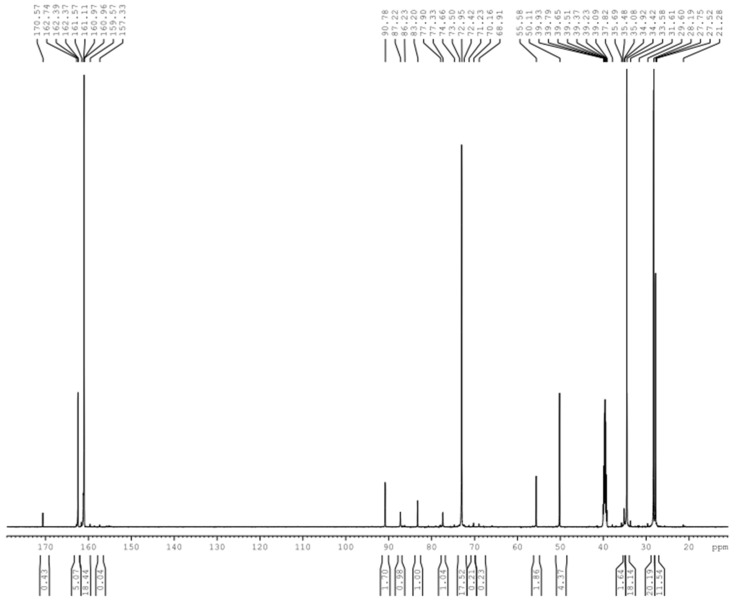
The ^13^C-NMR spectrum of sample A1.

Figure 1 shows the possible products formed in the DMUF reactions. Figure 2 shows the ^13^C-NMR spectrum of the sample under alkaline condition (A1). By referencing the literature [21], the chemical shifts were assigned to different types of methylene carbons and the quantitative results (relative contents) were list in Table 1. Due to the methyl groups on DMU are not directly involved in the reactions, only the chemical shifts were given in Figure 1, and quantitative calculations were not carried out.

As it can be seen from the quantitative results for sample A1 in Table 1, the hydroxymethylation of DMU is the primary reaction under alkaline conditions since the methylene carbon in hydroxymethyl group (71–73 ppm) accounts for 77.2%, and 16.3% formaldehyde in different forms remained unreacted. The signal at 61–62 ppm is completely absent in this sample, suggesting that the methylene linkage (structure D in Figure 1) was not formed. The condensation reactions produced, exclusively, the methylene ether linkage (structure E in Figure 1) at 77–78 ppm, but the products are minor as the methylene carbons account for only 4.6%, which may also include the contribution from the hemiformals formed through the addition between DMU and formaldehyde. These results indicate that the condensation reactions of DMU monomers are very slow indeed. In addition to the relatively lower temperature, another reason is that the condensations between DMU monomers can only form dimers. Nevertheless, the features of low reaction rate, predominant hydroxymethylation, and exclusive formation of ether linkages are similar to that of UF [22,23]. Previous theoretical calculations on base-catalyzed UF reactions propose an S_N_2 mechanism for the condensations between the UF monomers where the urea or hydroxymethylurea anions acted as reactive intermediates [23]. The energy barriers for both ether and methylene linkages were predicted to be higher than 100 kJ/mol, but the formation of ether linkages is energetically more favorable. Therefore, DMUF and UF reactions should share common mechanisms. The peak at 68–70 belongs to methylene carbons in the triazine ring [21], suggesting that a slight hydrolysis of DMU and recombination of the hydrolysis products occurred.

For the base-catalyzed melamine-formaldehyde (MF) reactions, our previous experiments [24] indicate that at pH = 9–10 condensations also dominantly formed ether bonds. Then a question arises: Why can MF reactions under alkaline produce resin, while UF cannot? The difference between melamine and urea, in structure, should be responsible for this. Melamine has three amino groups located in the rigid triazine ring with *meta* positions, therefore, even only unbranched ether linkages were formed, the polymers bear dimensional branching structure. However, UF polymers would have only linear structure if only ether linkages are formed and the cross-linked network will not be formed during the curing process. Thus, even though co-condensation reactions between MF and UF units under alkaline condition, only ether linkages can be formed, while such linkages in UF polymers are thought to be unstable toward hydrolysis.

**Figure 3:**
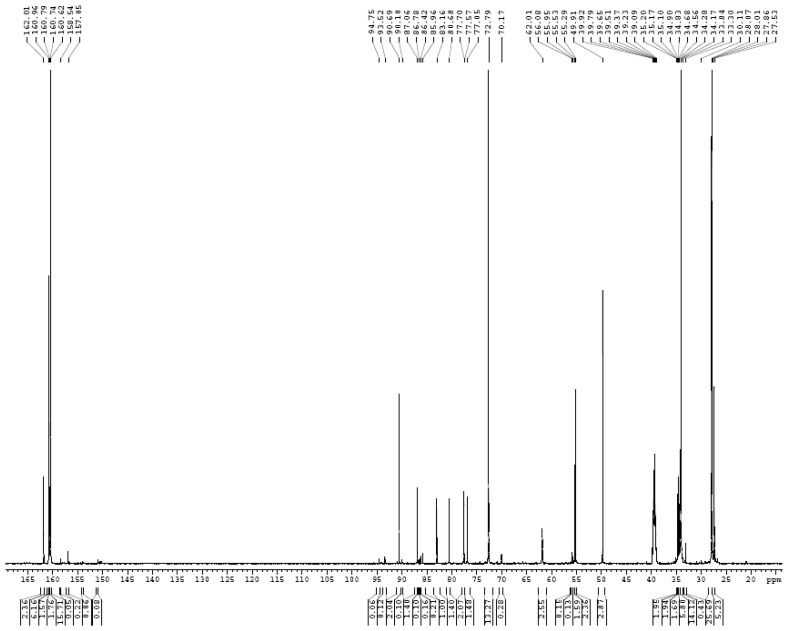
The ^13^C-NMR spectrum of sample A2.

**Figure 4:**
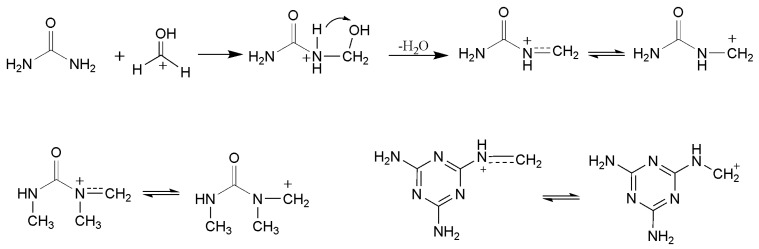
Formation of carbocation intermediates.

**Figure 5:**
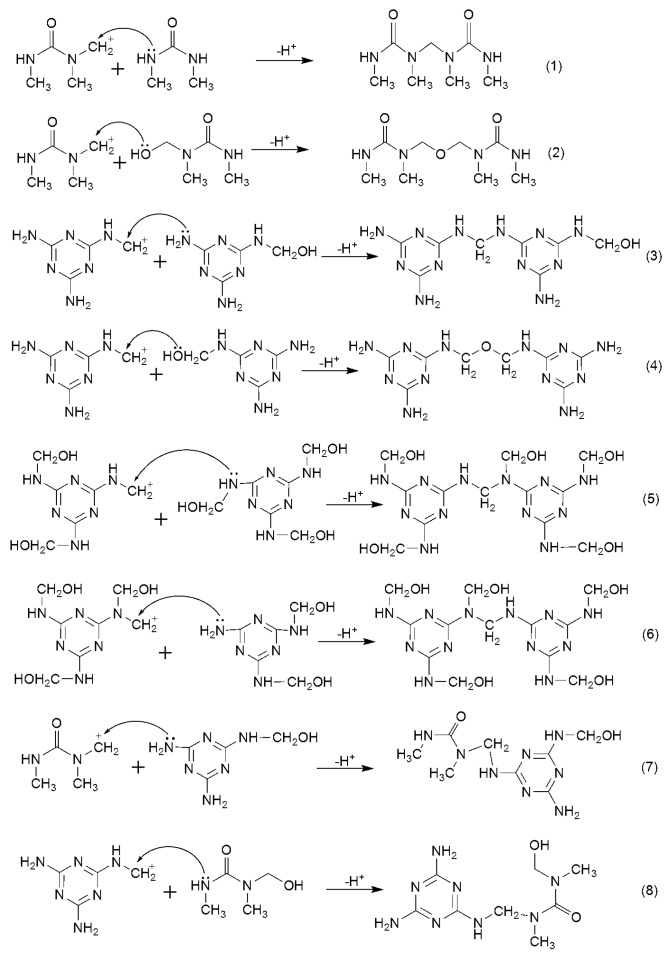
The representative acid-catalyzed condensation reactions.

Under acidic conditions, a very different spectrum was obtained, as shown in Figure 3. As it can be seen from the quantitative results for sample A2 in Table 1, the total content of methylene and ether linkage carbons was up to 23.2%, indicating that the condensation reactions appeared to be much faster. As shown in Figure 4, our previous theoretical calculations predicted that the carbocation intermediate can be produced through protonation of hydroxymethyl group and the condensation reactions were highly accelerated [25]. The calculated energy barriers suggested the formation of carbocation is a rate-determining step and represents an S_N_1 mechanism. Similar carbocation intermediates were also suggested for DMUF and MF reactions. The following representative condensation reactions were shown in Figure 5. If most of DMU molecules were hydroxymethylated at the beginning, then ether linkage should has been dominantly formed since DMU has only one reactive site. Note that the content of the methylene carbon in ether bond (13.5%) is higher than that (9.7%) of methylene linkage carbon (61–62 ppm). However, considering one ether linkage contains two carbons, while one methylene linkage contains one, the latter is more competitive, indeed. This result reflects hydroxymethylations and condensations occurred simultaneously under acidic conditions. The ^13^C-NMR tracking on acid-catalyzed UF reactions indicated that a part of ether linkages formed at the initial stage converted to a methylene linkage due to the latter being thermodynamically more stable and, finally, the methylene linkages became dominant [22,25]. In this context, the DMUF condensations here are still at the initial stage, and the methylene linkage will also become dominant if the reaction is allowed for a longer time.

### 3.2. The Melamine-Formaldehyde (MF) Reactions

**Figure 6:**
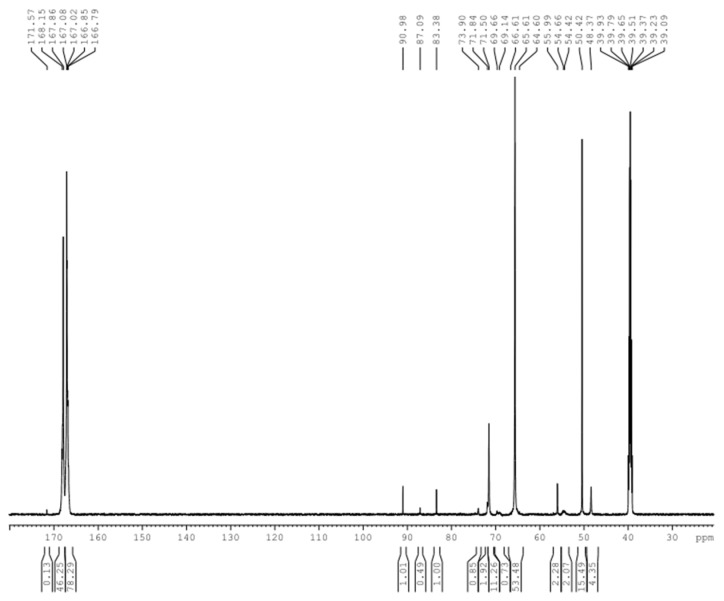
The ^13^C-NMR spectrum of sample B.

To compare with the DMUF system, the acid-catalyzed melamine-formaldehyde (MF) reactions were also investigated. The representative condensation reactions are listed in Figure 5 as reactions (3)–(6). The ^13^C-NMR spectrum of sample B was shown in Figure 6 and the corresponding quantitative results were also collected in Table 1. Compared with the results of DMUF, the total content of methylene carbons in this MF sample is much lower. However, this does not mean MF reactions are slower because the reactants were diluted in order to avoid fast gelation, as it has been described in the experimental procedures. It seems that MF reactions are quite different from DMUF reactions because the carbons of two types of methylene linkages account for 8.3%, whereas the ether bond carbon only account for 1.0%. Therefore, condensations leading to methylene linkages are much faster than those leading to ether bonds. Further, the content of the linear methylene linkage at 47–48 ppm (type I) is two times that of the branched linkage (type II). These features indicate that the condensation reaction (3) in Figure 5, namely, the reaction between the free amino group (–NH_2_) and the hydroxymethyl group (–NHCH_2_OH), is much faster than reactions (4)–(6). This implies, again, that hydroxymethylation and condensations take place simultaneously. Although the F/M molar ratio was set to 2/1, amino groups were still excessive to formaldehyde. Therefore, once a part of melamine molecules were hydroxymethylated, condensations began between un-reacted amino groups and hydroxymethyl groups. In other words, reaction (3) is statistically much more favorable than reaction (4) due to the presence of a large portion of free amino groups. As for reactions (5) and (6), they occur dominantly only when the F/M molar ratio is 3/1 or higher because only excessive formaldehyde can make the melamine highly hydroxymethylated. However, under this condition, formation of ether linkages will become much more competitive. Apparently, the acid-catalyzed MF reactions were totally different from the base-catalyzed reactions because different mechanisms are at work as different catalysts are used.

### 3.3. The Melamine-DMU-Formaldehyde (MDMUF) Reactions

After investigating the DMUF and MF reactions separately, the MDMUF reactions were then examined. The F/DMU/M molar ratio used here is 1/1/0.5 and this is the one being screened. Under alkaline conditions, for all molar ratios, the samples were always clear, but under acidic conditions, the molar ratios, like 2/1/1 and 1/1/1, caused fast gelation. With 1/1/0.2, ^13^C-NMR gave very weak MF signals. Among the tried molar ratios, only 1/1/0.5 gave clear samples and satisfied signals.

**Figure 7:**
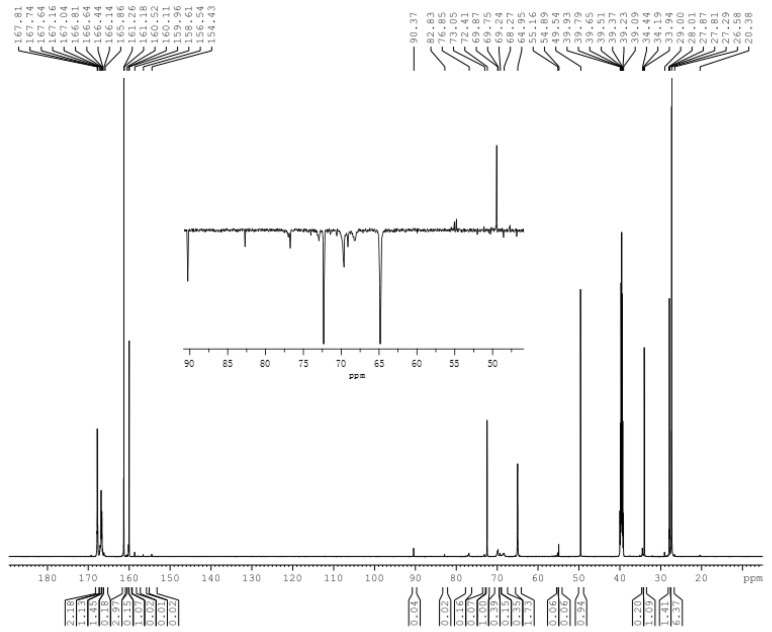
The ^13^C-NMR spectrum of sample C1.

The spectrum of sample C1 under alkaline conditions was shown in Figure 7 and quantitative results were given in Table 2. If co-condensation reactions occur, two possible ^13^C-NMR signals should appear. One is 54–55 ppm, which belongs to condensed methylene, but this peak may overlap with the methoxy structure –OCH_3_. Another is the 76–78 ppm that belongs to ether bonds. It can be seen in Figure 7 that two small peaks at 54.89 and 55.16 ppm appeared, indeed. However, the Dept135 spectrum confirmed that they belong to the methyl carbon of –O–CH_3_. The content of the carbon at 76–78 ppm accounts for 4.0%. However, self-condensation of a –NH–CH_2_OH group and a –N(–CH_2_OH)_2_ group on melamine (type II), as well as self-condensation of two hydroxymethylated DMUs, can also produce the branched ether bonds at 76–78 ppm. Therefore, even if the co-condensation reactions occurred, the products are very minor. In contrast, the ether bond is dominantly formed as the content of the carbon at 68–70 ppm (type I) accounts for 22.8%, suggesting that the self-condensations of MF monomer are faster than other condensations. Therefore, it is doubtful that true MUF co-condensed resin can be obtained throughout alkaline conditions.

**Figure 8:**
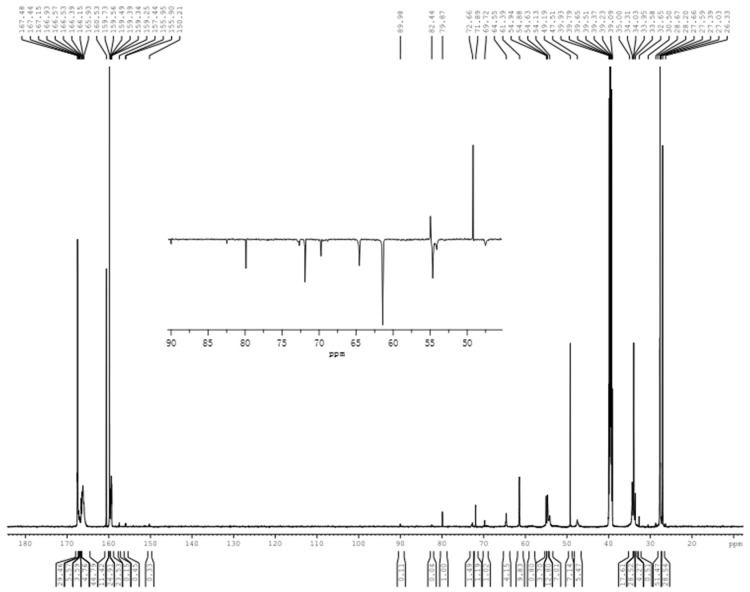
The ^13^C-NMR spectrum of sample C2.

Figure 8 shows the spectrum of the sample C2 of acid-catalyzed MDMUF reaction products and the quantitative results were also list in Table 2. It is easy to find that the results are very distinct from all of the situations above. Only 12.5% of the hydroxymethyl group and 0.4% of the formaldehyde remained unreacted, and 82.4% was converted to condensed methylene carbons, indicating the condensation reactions occurred very quickly. The carbon from unbranched methylene linkage at 47–48 ppm accounts for 12.8%. This structure was produced from the condensation of hydroxymethyl group (–NH–CH_2_OH) of melamine with a free amino group, as shown in Figure 5 as reaction (3). Meanwhile the self-condensation of hydroxymethylated DMU resulted in the branched methylene linkage at 61–62 ppm and the content of methylene carbon is 23.1%. It should be noted that the content of this type of carbon in DMUF reactions under acidic conditions is only 9.7%. This difference implies that the self-condensation of DMU monomers in the DMUF system is much slower than that in the MDMUF system. Another difference is the content of ether bond (68–70 ppm). In DMUF self-condensation reactions (sample A2), the ether bond carbon has a higher content than that of the methylene linkage, whereas in the MDMUF reactions, a very minor ether bond (2.4%) was formed. How does one understand these differences? With F:U:M = 1:1:0.5, a portion of melamine was hydroxymethylated. In other words, melamine consumed a part of formaldehyde. As a result, a large portion of DMU is unsubstituted and, therefore, the condensation occurred mainly between free DMU and hydroxymethylated DMU, resulting methylene linkage. This agrees with previous observations that a higher molar ratio favors formation of ether bonds, while a lower molar ratio favors formation of methylene linkages.

The most distinct change is that a very strong signal appeared at 54–55 ppm. The Detp135 spectrum indicates that most of the area of this broad peak belongs to the methylene carbon of the branched linkage =N–CH_2_–NH–. The quantitative result shows the content of this type of carbon accounts for 46.5%. According to the proposed mechanism, the reactions (5)–(8) in Figure 5 are the possible reactions that may lead to this type of linkage. The reactions (5) and (6) are the self-condensations of MF monomers. According to the results for the acid-catalyzed MF reactions (sample B in Table 1), with the F/M molar ratio of 2/1, the branched methylene linkage is only about half that of the linear linkage. In the MDMUF system, the F/DMU/M molar ratio was controlled to be 1/1/0.5. Under this condition, F/M molar ratio is much lower than 2/1 because DMU consumes a portion of formaldehyde. Therefore, condensations of MF monomers leading to branched linkages should be more minor. Since the linear linkage accounts for 12.8% in the MDMUF system, contributions from reactions (5) and (6) should be lower than 6%. Therefore, over 40% of the carbons at 54–55 ppm should be contributed by reactions (7) and (8). Although uncertainty exists in such inferential calculations, it should still be safe to conclude that the contribution from co-condensations is dominant. A remaining question here is why the co-condensation reactions were prior to the MF self-condensations. The plausible explanation is that the concentration of DMU is two times that of the concentration of melamine. As a result, the collisions between MF monomers with DMU monomers have higher probability than collisions among MF monomers.

In the real synthesis of MUF resin, the molar ratio of the start reactants should be another factor that influences of the structures of the polymers. Specifically, the F/(U + M) molar ratio determines the relative content of methylene and ether linkages, while the U/M molar ratio affects the relative content of co-condensed and self-condensed structures. Therefore, it is also of importance to balance the cost, performance, and operability in resin synthesis. In summary, the present work provided an indisputable result that the pH (or catalyst) is the key factor that determines whether co-condensation can occur efficiently, although the experiments are simple.

Although this study clearly indicated that the MUF co-condensation cannot occur, or occur to a very limited extent under alkaline conditions, it cannot be ruled out that co-condensations can occur during the curing process of resin because acidic catalysts, such as NH_4_Cl and (NH_4_)_2_SO_4_, are generally used to accelerate the curing process. However, within a short hot-press time (generally several minutes), it is doubtful that the co-condensation reactions would be completed. To clarify this issue, it is necessary to investigate the cured resin structure.

## 4. Conclusions

In this study, the 1,3-dimethylurea was used to model MUF reactions. The reaction products of DMUF, MF, and MDMUF under alkaline and weak acidic conditions were compared by performing quantitative ^13^C-NMR analysis. The effect of pH on the co-condensation reactions was clarified.

It was found that DMUF condensations were very slow and hydroxymethylation appeared to be primary at pH = 9.0–9.5. In contrast, MF condensations appeared to be faster even under alkaline conditions, but only ether linkages were formed. Under acidic condition (pH = 6.0–6.3), both DMUF and MF condensations became faster and methylene linkages were efficiently formed.

With the presence of the methyl groups in DMU, the appearance or absence of the featured signal at 54–55 ppm can be used to identify the co-condensed methylene linkage –N(–CH_3_)–CH_2_–NH. At pH = 9.0–9.5, MDMUF reactions produced mainly MF polymers and the featured signal at 54–55 ppm was absent. Even though the co-condensations occurred, indistinguishable and very minor condensed structures with ether linkage were formed. Differently, under weak acidic conditions (pH = 6.0–6.3), a very strong signal at 54–55 ppm appeared and the relative content of methylene carbons accounts for over 40%, indicating the MDMUF co-condensation reactions were much more competitive than the self-condensations. The formation of a highly-reactive carbocation intermediate was proposed to rationalize the results.

## Figures and Tables

**Table 1:** ^13^C-NMR assignment and quantitative results (%) for DMUF and MF samples.

F:DMU = 1:1				F:M = 2:1		
Structures	Chemical shift (ppm)	A1	A2	Structures	Chemical shift (ppm)	B
		pH = 9	pH = 6			pH = 6
–N(CH_3_)–CH_2_–N(CH_3_)–	61–62	—	9.7	–NH–CH_2_–NH– (I)	47–48	5.6
Triazine(–CH_2_–)	68–70	1.9	1.1	–NH–CH_2_–N= (II)	54–55	2.7
H_3_C–NH–CO–N(–CH_3_)–CH_2_OH	71–73	77.2	50.6	=N–CH_2_–N= (III)	60–61	——
					**Total**	**8.3**
–N(CH_3_) –CH_2_–O–CH_2_–N(CH_3_)- –N(–CH_3_)–CH_2_OCH_2_OH	77–78	4.6	13.5			
				–NH–CH_2_OCH_2_NH– (I)	68–70	1.0
				–NH–CH_2_OCH_2_N= (II)	77–78	——
Uron(–CH_2_–O–CH_2_–) –N(–CH_3_)CH_2_OCH_3_	80–81	—	5.3			
					**Total**	**1.0**
	**Total**	**83.7**	**80.2**	–NH–CH_2_OH (I)	64–66	69.3
HO–CH_2_–OH	82–84	4.4	3.8	–N(–CH_2_)–CH_2_OH (II)	71–73	17.1
HOCH_2_–O–CH_2_–OCH_2_OH –N(–CH_3_)–CH_2_–O–CH_2_OH	86–88	4.4	7.1		**Total**	**86.4**
				HO–CH_2_–OH	82–84	1.3
HOCH_2_-O–CH_2_–OCH_2_OH	90–91	7.5	8.2	HOCH_2_-O–CH_2_–OCH_2_OH –NH–CH_2_–O–CH_2_OH	86–88	0.6
H(CH_2_O)_n_OCH_2_OCH_3_	93–95	—	0.7	HOCH_2_–O–CH_2_–OCH_2_OH	90–91	1.3
	**Total**	**16.3**	**19.8**	H(CH_2_O)_n_O–CH_2_OCH_3_	93–95	——
					**Total**	**3.2**
				–NH–CH_2_–O–CH_3_	73–74	1.1

**Table 2:** ^13^C-NMR assignment and quantitative results (%) for MDMUF samples.

Structures	Chemical Shift (ppm)	C1	C2	Structures	Chemical Shift (ppm)	C1	C2
		pH = 9	pH = 6			pH = 9	pH = 6
–NH–CH_2_–NH– (I)	47–48	——	12.8	–NH–CH_2_OH (I)	64–66	44.3	9.7
	54–55	——	46.5		71–73	25.6	2.8
–NH–CH_2_–N= (II)				–N(–CH_2_) –CH_2_OH (II)			
–NH–CH_2_–N(–CH_3_)–				–N(–CH_3_) –CH_2_OH			
	61–62	——	23.1		**Total**	**69.8**	**12.5**
=N–CH_2_–N= (III)							
–N(–CH_3_) –CH_2_–N(–CH_3_)-				HO–CH_2_–OH	82–84	0.4	0.1
	**Total**	**0**	**82.4**		86–88	——	——
				HOCH_2_–O–CH_2_–OCH_2_OH			
–NH–CH_2_OCH_2_NH– (I)	68–70	22.8	2.4				
				–N(–CH_3_) –CH_2_OCH_2_OH			
–NH–CH_2_OCH_2_N= (II)	76–78	4.0	——	HOCH_2_–O–CH_2_–OCH_2_OH	89–91	1.1	0.3
=N–CH_2_OCH_2_N= (III)				H(CH_2_O)_n_O–CH_2_OCH_3_	93–95	——	——
–N(–CH_3_)–CH_2_OCH_2_–N(-CH_3_)					**Total**	**1.5**	**0.4**
Uron(–CH_2_–O–CH_2_–)	78–80	——	2.3	–NH–CH_2_–O–CH_3_	73–74	1.9	——
–NH(–CH_3_) –CH_2_OCH_3_							
	**Total**	**26.8**	**4.7**				
